# People living with HIV display increased anti-apolipoprotein A1 auto-antibodies, inflammation, and kynurenine metabolites: a case–control study

**DOI:** 10.3389/fcvm.2024.1343361

**Published:** 2024-02-13

**Authors:** Miguel A. Frias, Sabrina Pagano, Nasim Bararpour, Jonathan Sidibé, Festus Kamau, Vanessa Fétaud-Lapierre, Peter Hudson, Aurélien Thomas, Sandrine Lecour, Hans Strijdom, Nicolas Vuilleumier

**Affiliations:** ^1^ Division of Laboratory Medicine, Diagnostic Department, Geneva University Hospitals, Geneva, Switzerland; ^2^ Department of Medical Specialties, Faculty of Medicine, University of Geneva, Geneva, Switzerland; ^3^ Faculty Unit of Toxicology, CURML, Faculty of Biology and Medicine, University of Lausanne, Lausanne, Switzerland; ^4^ Department of Genetics, Stanford University, Stanford, CA, United States; ^5^ Stanford Center for Genomics and Personalized Medicine, Stanford, CA, United States; ^6^ Centre for Cardiometabolic Research in Africa, Division of Medical Physiology, Faculty of Medicine and Health Sciences, Stellenbosch University, Cape Town, South Africa; ^7^ Cape Heart Institute, Department of Medicine, Faculty of Health Sciences, University of Cape Town, Cape Town, South Africa; ^8^ Unit of Forensic Toxicology and Chemistry, CURML, Lausanne and Geneva University Hospitals, Lausanne, Geneva, Switzerland

**Keywords:** HIV, anti-retroviral therapy, autoimmunity, anti-apolipoprotein A1 auto-antibodies, cardiovascular disease, kynurenine pathway metabolites

## Abstract

**Objective:**

This study aimed to study the relationship between auto-antibodies against apolipoprotein A1 (anti-apoA1 IgG), human immunodeficiency virus (HIV) infection, anti-retroviral therapy (ART), and the tryptophan pathways in HIV-related cardiovascular disease.

**Design:**

This case–control study conducted in South Africa consisted of control volunteers (*n* = 50), people living with HIV (PLWH) on ART (*n* = 50), and untreated PLWH (*n* = 44). Cardiovascular risk scores were determined, vascular measures were performed, and an extensive biochemical characterisation (routine, metabolomic, and inflammatory systemic profiles) was performed.

**Methods:**

Anti-apoA1 IgG levels were assessed by an in-house ELISA. Inflammatory biomarkers were measured with the Meso Scale Discovery® platform, and kynurenine pathway metabolites were assessed using targeted metabolomic profiling conducted by liquid chromatography-multiple reaction monitoring/mass spectrometry (LC-MRM/MS).

**Results:**

Cardiovascular risk scores and vascular measures exhibited similarities across the three groups, while important differences were observed in systemic inflammatory and tryptophan pathways. Anti-apoA1 IgG seropositivity rates were 15%, 40%, and 70% in control volunteers, PLWH ART-treated, and PLWH ART-naïve, respectively. Circulating anti-apoA1 IgG levels were significantly negatively associated with CD4+ cell counts and positively associated with viremia and pro-inflammatory biomarkers (IFNγ, TNFα, MIPα, ICAM-1, VCAM-1). While circulating anti-apoA1 IgG levels were associated with increased levels of kynurenine in both control volunteers and PLWH, the kynurenine/tryptophan ratio was significantly increased in PLWH ART-treated.

**Conclusion:**

HIV infection increases the humoral response against apoA1, which is associated with established HIV severity criteria and kynurenine pathway activation.

## Introduction

Sub-Saharan Africa bears the highest burden of human immunodeficiency virus (HIV) globally (1). South Africa has the largest population of people living with HIV (PLWH), estimated at 6 million people living with the virus (which represents 12% of the total South African population). Thanks to the availability of improved treatments, such as anti-retroviral therapy (ART), HIV is no longer considered a fatal illness. Therefore, the clinical challenges confronting the HIV population have now shifted from dealing with acquired immunodeficiency syndrome (AIDS)-related illnesses to managing chronic diseases, such as cardiovascular disease (CVD).

It is now established that the risk of developing CVD is significantly elevated, accelerated, and associated with poorer outcomes in PLWH compared to the general population. In developed countries, HIV-infected patients experienced a two- to six-fold increase in coronary artery disease that occurs at an earlier stage compared to the non-infected population (2, 3). In addition, higher mortality rates after a first myocardial infarction are observed in HIV patients (4). Multiple factors potentially contribute to the pathophysiology of HIV-related CVD and include the HIV itself, adverse effects of ART, and processes such as inflammation, immune/autoimmune activation, endothelial injury, and disordered coagulation. These features may contribute to the increase in HIV-related cardiovascular risk (5).

The mechanisms by which HIV and ART favour CVD have yet to be fully elucidated, and the delineation of biomarkers to assess cardiovascular risk in the HIV population at an early stage is required to limit the public health burden. Among potential candidate biomarkers and mediators that could causally be linked to infections, immune dysregulation, and subsequent non-communicable diseases (including CVD), the kynurenine pathway is gaining notable momentum (6–9). Pathogens, including HIV, as well as the cytokine host response, are known to activate the kynurenine pathway, subsequently affecting host immune tolerance and overall gut, vascular, and brain inflammatory responses. Activation of the kynurenine pathway is therefore suspected to negatively modulate the course of a broad range of pathologies, including infections, tumoral, cardiovascular, and neurological diseases (10–12). Recently, the kynurenine pathway has also been shown to be involved in uncontrolled B-cell activation, leading to the production of auto-antibodies (6), a biological signature often observed in HIV patients (13, 14).

Among auto-antibodies produced in the context of different RNA viral infections and of possible relevance for CVD, those directed against apolipoprotein A1 (anti-apoA1 IgG), the major protein fraction of high-density lipoprotein (HDL), are of particular interest (15–17). Growing evidence from studies involving hepatitis C, HIV, and SARS-CoV2 infections (18–20) indicates that anti-apoA1 IgG may serve as a mediator of atherogenesis through definite innate immune receptor signalling *in vivo* (21–24) and as a biomarker predicting worse cardiovascular outcomes in different human pathologies according to numerous prospective longitudinal cohort studies [reviewed in (25)]. In a recent study involving the Swiss HIV cohort, high anti-apoA1 IgG levels were found to be associated with low CD4+ cell counts, high viremia, and a pro-inflammatory systemic profile and could promote CD4+ lymphocyte apoptosis (19). All biological effects of anti-apoA1 IgG are mediated by its binding to TLR2, which elicits the formation of the TLR2/TLR4/CD14 complex, triggering pro-inflammatory and pro-atherogenic responses through NF-κB and AP-1 pathways (19, 22, 26).

However, there is currently no information linking the HIV-induced anti-apoA1 IgG response to tryptophan/kynurenine metabolism. Therefore, in the present study, we aimed to determine the links between the HIV-induced anti-apoA1 IgG response and kynurenine metabolism, inflammation, cardiovascular risk, and ART using a unique cohort of PLWH ART-treated and ART-naïve from South Africa.

## Materials and methods

### Participants

Blood samples were collected from 144 participants recruited in the study “EndoAfrica” (27). Patients were recruited upon presentation at HIV clinics or community health centres in Cape Town, South Africa. On the day of recruitment, participants underwent procedures, including informed consent, completion of a health questionnaire, measurement of body mass index, waist circumference, and waist–hip ratio, assessment of blood pressure and heart rate, provision of a urine sample, collection of fasting blood, and examination of flow-mediated dilatation (FMD) and carotid intima-media thickness (cIMT); most of the clinical characteristics were also assessed on the day of recruitment. The Table 1 is divided into groups: control volunteers (*n* = 50), PLWH on ART (PLWH ART+) (*n* = 50), and untreated PLWH (PLWH ART−) (*n* = 44). Patients were matched between groups for age and sex. The number of patients taking statins was not significant, as only one patient was under statins (simvastatin) at the time of blood collection. This patient belongs to the PLWH ART– group. Informed written consent was obtained from all the participants. The study adhered to the ethical guidelines of the Declaration of Helsinki and was reviewed and cleared by the Ethical Committee of the University of Stellenbosh (South Africa).

PLWH included in this study were over 18 years old, not pregnant, and over 3 months post-partum. PLWH on ART were treated with efavirenz 600 mg + tenofovir DF 300 mg + emtricitabine 200 mg (*n* = 46), Nevirapine 200 mg (NVP)/lamivitudine 150mg + zidovudine 300 mg (Lamzid) (1), Lamivudine/Efavirenz/Abacavir (1), and Ritonavir 50 mg + lopinavir 200 mg (Aluvia)/Tenofovir/Lamivudine (1). The median duration of treatment was 176 weeks, with an interquartile range of 108–300 weeks.

PLWH-ART naïve showed a median time between HIV diagnosis and sampling of 65 days, with an interquartile range of 23.5–406 days (*n* = 32).

### Blood tube collections and biochemical parameters

A qualified research nurse conducted anthropometric measurements, urine collection, and phlebotomy. Anthropometric measurements, including height (cm) measured with a stadiometer, weight (kg) measured using an electronic scale, and waist and hip circumferences (cm) measured with a measuring tape, were performed per standardised protocols. Body mass index (BMI, kg/m^2^) and waist-to-hip ratio were calculated. Systolic blood pressure (SBP, mmHg) and diastolic blood pressure (DBP, mmHg) were measured thrice at 5-min intervals using an Omron M6 automatic digital blood pressure monitor (Omron Healthcare, Kyoto, Japan) around the left brachium. Subsequently, the average value was calculated. Study participants fasted from 10:00 pm the night before clinical assessments. Participants with unknown HIV status were tested for HIV using a rapid HIV test (SD Bioline HIV 1/2 3.0 immunochromatographic kit; Standard Diagnostics, Republic of Korea) to determine their HIV status. Urine and fasting blood samples were collected and sent to the National Health Laboratory Service (NHLS) for biochemical analyses using standard laboratory techniques. Plasma lipid profiles [total cholesterol (TC), high-density lipoprotein cholesterol (HDL-C), low-density lipoprotein cholesterol (LDL-C), and triglyceride (TG) levels, mmol/L] were determined using a chemiluminescence methodology (cobas® 301/501 analyser, Roche/Hitachi cobas® c systems, Basel, Switzerland). Levels of liver enzymes [gamma-glutamyl transferase (GGT, U/L) and alanine aminotransferase (ALT, U/L)] were determined via an enzymatic chemiluminescence methodology using a cobas® 311/501 analyser (Roche/Hitachi cobas® c systems, Basel, Switzerland). Levels of high-sensitivity C-reactive protein (hsCRP, mg/L) were obtained via an IMMAGE® Immunochemistry Systems and Calibrator 5 Plus assay kit (Beckman Coulter, Inc., CA, USA). Fasting glucose levels (mmol/L) and glycated haemoglobin (HbA1C, Hb%) were determined using a chemiluminescence methodology (haemolysate application on the cobas® 311/501 analyser, Roche/Hitachi cobas® c systems, Basel, Switzerland). Haemoglobin (Hb, g/dL) levels were determined using a chemiluminescence method (whole blood application on the cobas® 311/501 analyser, Roche/Hitachi cobas® c systems, Basel, Switzerland). Urine samples were analysed to determine microalbuminuria (mg/L) and creatinine (mmol/L) using an enzymatic chemiluminescence method by cobas® 501/502 and cobas® 311/501 analysers (Roche/Hitachi cobas® c systems, Basel, Switzerland), respectively, and the albumin-to-creatinine ratio (ACR) (mg/mmol) was computed. In HIV+ participants, the levels of cluster of differentiation four (CD4)+ T-cell count and viral load (VL) were determined by flow cytometry (FC 500 MPL) with MXP software (Beckman Coulter, Brea, CA, USA) and the COBAS® AmpliPrep/COBAS® TaqMan® HIV-1 Test, v2.0 (Roche Diagnostics Ltd., Burgess Hill, UK), respectively (28).

Blood samples were collected in Becton Dickinson vials to obtain serum or EDTA plasma. After collection, the serum was allowed to coagulate at room temperature for 30 min. Serum and plasma samples were centrifuged at 2,300g for 5 min at room temperature, aliquoted, and stored at −80°C until further analyses. Apolipoproteins A1 and B were analysed by a Cobas e501 automated system using electrochemiluminescence technology from Roche Diagnostics (Roche, Rotkreuz, Switzerland) at the University Hospital of Geneva, Geneva, Switzerland.

### Cardiovascular event risk estimation

Cardiovascular risk was assessed using the 10 years Framingham risk score (FRS) calculation, the extent of carotid atherosclerotic vascular disease was evaluated using the carotid intima-media thickness (cIMT) measurement, and the endothelial function was assessed using the flow-mediated dilation (FMD) measurement.

The FRS was calculated using the calculator prepared by R.B. D'Agostino and M.J. Pencina based on a publication by D'Agostino et al. (29). FRS calculation is based on gender, age, systolic blood pressure, treatment for hypertension, smoking, presence of diabetes, total cholesterol, and HDL cholesterol.

FMD assessment was performed using a MyLab™ Five mobile ultrasound system (Esaote, Italy). The FMD protocol followed previously published recommendations (30, 31). Participants were positioned supine on an examination bed, with their right arm abducted and supinated. A blood pressure cuff (deflated) was placed around the proximal part of the forearm. Subsequently, the ultrasound probe was positioned proximal to the cubital fossa (mid to distal humerus), just below the biceps brachii muscle belly, until the brachial artery was visually located on the ultrasound image. The ultrasound probe was then secured in this position using a probe holder. Next, cross-sectional still images were captured with the ultrasound probe at three different locations along the designated section of the artery to obtain baseline brachial artery diameter measurements. Following this, the blood pressure cuff was inflated to 200 mmHg (or 50 mmHg supra-systolic in the case of individuals with systolic blood pressure greater than 150 mmHg), and blood flow to the forearm was occluded for 5 min. After the blood pressure cuff was deflated, additional cross-sectional ultrasound stills were taken along the same section of the artery for a duration of 2 min. For still analysis, the built-in manual measurement tool of the MyLab™ Five mobile ultrasound system was used to measure the brachial artery diameter in millimetres, consistently at the end of diastole, in all the stills. The three baseline measurements were used to calculate a mean baseline brachial artery diameter, and the maximum post-occlusion measurement, usually at approximately 60 s after blood pressure cuff release, was used to calculate the FMD percentage according to the following formula:

$$\mathrm{FMD}\;\text{\%}\;=\;\frac{\mathrm{maximum}\;\mathrm{post}\text{-}\mathrm{occlusion}(\mathrm{diameter}(\mathrm{mm})-\mathrm{mean}\;\mathrm{baseline}\;\mathrm{diameter}(\mathrm{mm}))}{\mathrm{mean}\;\mathrm{baseline}\;\mathrm{diameter}(\mathrm{mm})}\times 100$$

To ensure reliable data collection, the ultrasound operators were subjected to stringent training by experts and multiple practice sessions with student volunteers and colleagues.

To keep inter-operator variability to a minimum, only three trained and experienced operators were employed in this study, and image analysis and data acquisition were performed independently by a single person (32).

Of note, for statistical correlation tests, the FMD values used for correlation analysis included negative values. To overcome negativity, four was added to the FMD value to shift them from negative value and then the values were log2-transformed.

The carotid intima-media thickness (cIMT) was measured using an Esaote MyLab Five portable ultrasound device (Genoa, Italy) equipped with an Esaote Doppler probe (LA523, 12 MHz) and QIMT software, which automatically and accurately measures all the parameters needed for carotid IMT measurements. For the IMT protocol, participants were asked to lie in a supine position with their head resting comfortably and their neck slightly hyperextended and tilted 45° to the opposite side of the carotid artery being assessed. The operator (author of this dissertation) used the index and middle fingers to locate the pulsating carotid artery to guide the position of the probe on the participant's neck. The position of the probe was further adjusted until a clear and stable image was obtained on the ultrasound. The lateral angle of the image was assessed as far as possible. In cases where a lateral view was not optimal (clear), an anterior or posterior angle was used. Once the image was clear and stable, the region of interest was manually placed 5 mm proximal to the dilatation of the carotid bulb. Thereafter, QIMT software automatically detected and analysed the vascular boundaries in radio frequency (RF) mode. The software further calculated the diameter and the thickness of the intima-media layer, as well as the median and standard deviation IMT values, using high spatial resolution. At this stage, the IMT measurements were also calculated and expressed in micrometres (μm), with the standard deviation changing. Once the standard deviation fell below 25 μm (the value also turns green), the IMT image was frozen and saved. These automatic and accurate measurements are largely independent of the investigator and the device settings. For this study, both the left and right common carotid arteries were assessed, and values from both sides were averaged and used in data analysis.

### Inflammatory cytokine assessment

All inflammatory biomarker analyses were performed on serum samples at the Geneva University Hospitals. Inter-cellular adhesion molecule-1 (ICAM-1), vascular cell adhesion molecule-1 (VCAM-1), interleukin (IL)-6, -8, -10, interferon (INF)-gamma, tumour necrosis factor (TNF)-alpha, macrophage inflammatory protein 1-alpha and -beta (MIP1 alpha and MIP1 beta), serum amyloid A (SAA) and monocyte chemoattractant protein-1 (MCP-1), were measured using the Meso Scale Discovery (MSD) platform (Rockville, MD, USA). Analyte concentrations were determined by Discovery Workbench® software 4.0 using a four-parameter logistic fit model. The lower limits of detection in pg/mL were as follows: IL-6 and IL-8, 0.04; IL-10, 0.03; INFγ, MIP1 alpha, MIP1 beta, SAA, TNF-α, 0.04; and MCP-1, 0.09. Intra-run CVs were below 7%, and inter-run CVs were below 15%.

### Assessment of anti-apoA-1 IgG levels

Anti-apoA-1 IgG levels were measured as previously described (17, 23, 33). Briefly, MaxiSorp plates (NuncTM, Denmark) were coated with purified, human-derived delipidated apolipoprotein A-1 (20 μg/mL; 50 μL/well) for 1 h at 37°C. After washing, all wells were blocked for 1 h with 2% bovine serum albumin (BSA) in phosphate-buffered saline (PBS) at 37°C. Patient samples were also added to a non-coated well to assess individual non-specific binding. After six washing cycles, 50 μL/well of signal antibody (alkaline phosphatase-conjugated anti-human IgG; Sigma-Aldrich, St. Louis, MO, USA), diluted 1:1,000 in a PBS/BSA 2% solution, was added and incubated for 1 h at 37°C. After six more washing cycles, phosphatase substrate p-nitrophanylphosphate disodium (Sigma-Aldrich) dissolved in diethanolamine buffer (pH 9.8) was added and incubated for 30 min at 37°C (Molecular Devices TM Filtermax 3). The optical density (OD) was determined at 405 nm, and each sample was tested in duplicate. Corresponding non-specific binding was subtracted from the mean OD for each sample. The specificity of detection was assessed using conventional saturation tests by western blot analysis. As previously described, elevated levels of anti-apoA-1 IgG (seropositivity) were defined by an OD cut-off of OD ≥0.64 and a ratio between the sample net absorbance and the positive control net absorbance × 100 above 37, corresponding to the 97.5th percentile of a reference population.

### Human aortic endothelial cells (HAECs)

HAECs were purchased from Ruwag (Bettlach, Switzerland). Briefly, HAECs were grown in a complete EBM-2 medium supplemented with 5% FBS (foetal bovine serum). HAECs were stimulated in EBM-2 medium supplemented with 0% FBS for 24 h without or with polyclonal goat anti-apoA1 IgG (Academy Bio-Medical Co) (40 µg/mL) or polyclonal goat IgG (Meridian Life Science) (40 µg/mL). The protocol was chosen based on previous evaluations of the optimal time course for inducing a pro-inflammatory response, which determined that 24 h of stimulation with anti-apoA1 IgG at a concentration of 40 µg/mL yielded better results (21–24, 26, 33, 34). Afterwards, cells were collected, and RNA was isolated and analysed using a LightCycler 480 Real-Time PCR System (Roche) in 96-well plates. The amplification curves were analysed using Roche LightCycler software to determine Cp (by the second derivative method). Primers for human ICAM-1 (Hs 00164932_m1), VCAM-1 (Hs 01003372_m1), and GAPDH (Hs 99999905_m1) as housekeeping genes were used and purchased from Thermo Fisher (Basel, Switzerland). The stimulating medium was collected, and ICAM-1 and VCAM-1 were quantified using the Meso Scale Discovery platform, as described above. In the same supernatant, the levels of kynurenine, tryptophan and kynurenic acid were measured using liquid chromatography-multiple reaction monitoring/mass spectrometry (LC-MRM/MS).

### Kynurenine pathway metabolomics

Metabolites were extracted using a methanol–ethanol solvent mixture in a 1:1 ratio. After protein precipitation, the supernatant was evaporated to dryness and finally resuspended with 100 µL of 10% methanol (MeOH) in H_2_O. The samples were analysed by LC-MRM/MS on a hybrid triple quadrupole-linear ion trap (QqQ_LIT_) system (Qtrap 5500, Sciex) coupled to an LC Dionex Ultimate 3,000 (Dionex, Thermo Scientific). Analysis was performed in positive and negative electrospray ionisation modes using a TurboV ion source. The MRM/MS method included 299 and 284 transitions in positive and negative modes, respectively, corresponding to 583 endogenous metabolites. The chromatographic separation was performed on a Kinetex C18 column (100 × 2.1 mm, 2.6 µm). For the positive mode, mobile phase A consisted of 0.1% FA in H_2_O and mobile phase B consisted of 0.1% FA in acetonitrile (ACN). For the negative mode, mobile phase A consisted of 0.5 mM ammonium fluoride in H_2_O and mobile phase B consisted of 0.5 mM ammonium fluoride in ACN.

The linear gradient program was as follows: 0–1.5 min, 2% B; 1.5–15 min, up to 98% B; 15–17 min, held at 98% B; 17.5 min, down to 2% B, with the flow rate maintained at 250 µL/min.

The MS instrument was controlled by Analyst software v.1.6.2 (AB Sciex). Peak integration was performed with MultiQuant software v.3.0 (AB Sciex). The integration algorithm was MQ4 with a Gaussian smoothing of a half-width equal to 1.5 points.

Data obtained after MSTUS (MS Total useful signal) normalisation were used (35, 36). For better visualisation, a factor of 10^6^ was applied after normalisation.

### Statistics

Non-parametric Kruskal–Wallis and Mann–Whitney tests were used for assessing differences between groups, and Spearman tests were used for correlation analysis. In *in vitro* experiments with HAECs, paired Wilcoxon tests were used for analysing differences between groups. For metabolite analyses, the data were expressed as log-normalised. The correlation between anti-apoA1 IgG, cIMT, FMD, and metabolites was analysed using Spearman’s rank test. Differences between groups were tested using the Mann–Whitney test. *P* < 0.05 was considered as significant. Statistical analyses were performed using Prism 9 (GraphPad Prism, CA, USA).

## Results

### Population characteristics

**Figure 1:**
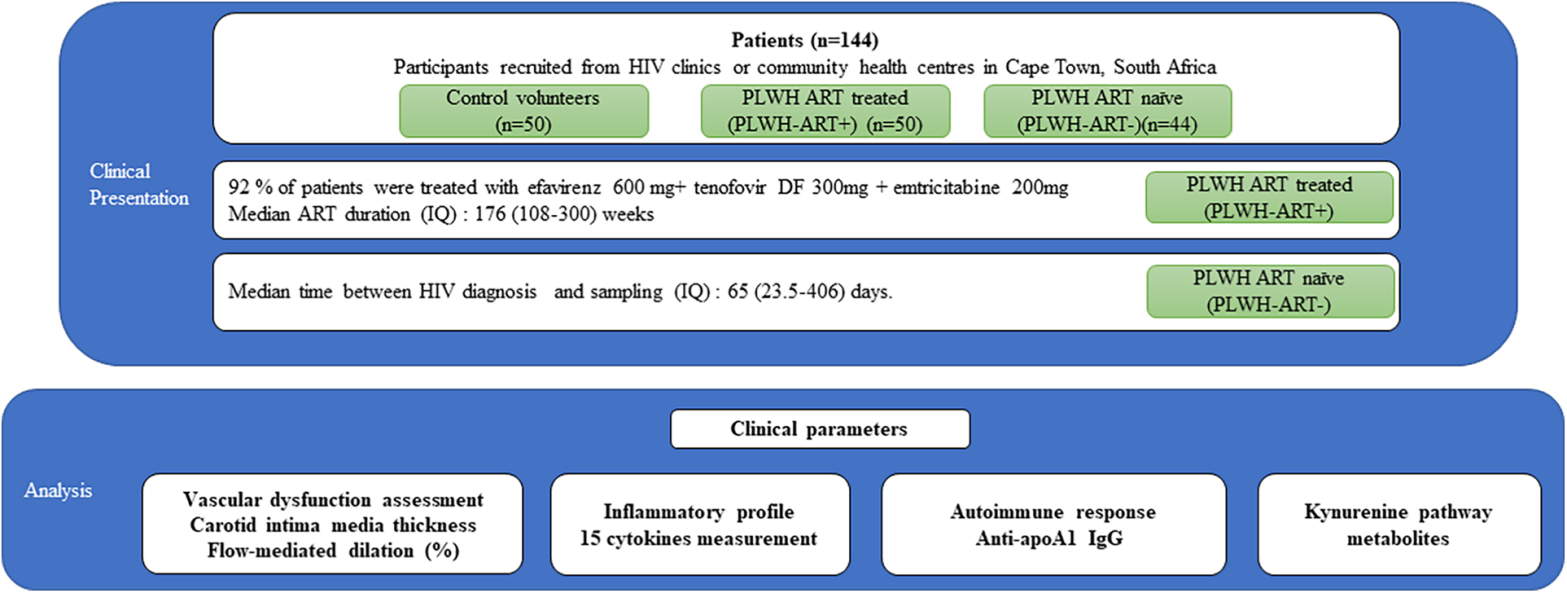
Study design. Clinical parameters and samples from 144 patients were analysed. These subjects were divided into three groups: control volunteers without HIV (*n* = 50), PLWH on ART (PLWH ART+) (*n* = 50), and PLWH ART-naïve (PLWH ART−) (*n* = 44). The analysis included clinical parameters, inflammatory profile, autoimmune response, and kynurenine pathway metabolites.

Participants were divided into three groups: HIV-free control volunteers (*n* = 50), HIV-positive patients on ART (*n* = 50), and HIV-positive ART naïve patients (*n* = 44) (Figure 1). Cardiovascular risk profile, subclinical atherosclerosis status, and endothelial dysfunction were assessed using FRS, cIMT, and FMD, respectively. The baseline demographic and clinico-biological characteristics of the 144 patients are summarised in Table 1. Across the three groups (healthy volunteers, PLWH ART-treated, and PLWH ART-naïve), we observed a decreasing trend for HDL-C and apoA1 levels, with the lowest levels observed in PLWH ART-naïve (Table 1), while increasing trends were observed for most markers of systemic inflammation measured in negative controls vs. the PLWH group, with a few exceptions. MIP-1 beta, MCP-1, and SAA levels were similar between the three groups. IL-8 and hsCRP were significantly increased in PLWH ART-treated compared to healthy volunteers, while we observed no differences between healthy volunteers and PLWH ART-naïve for these parameters (Table 1). Regarding the tryptophan pathway metabolites, similar trends were observed for most tryptophan metabolites across these three groups, with the kynurenine/tryptophan ratio being the highest in PLWH ART-naïve. Conversely, a decreasing trend was observed for xanthurenic acid across these groups (Table 1).

**Table 1:** Clinical parameters, CVD risk, inflammatory parameters, and tryptophan pathway metabolites in the different groups.

Parameters	Control volunteers	PLWH ART-treated	PLWH ART-naïve
*N*	50	50	44
Age	35 (31–42)	36 (33–45)	37 (28–44)
Gender (male/female)	16/33	15/35	13/31
HIV profile			
CD4+ (cells/µL)		517 (399–716.5)	452.5 (298.75–558.25)
Viral load (RNA copies/mL)		20 (10–53)	21,925 (4,602–68,569.5)^§§§§^
Metabolic profile			
BMI (kg/m^2^)	21.4 (19–27.7)	21.9 (18.9–28.1)	20.7 (18.425.55)
Waist/hip ratio	0.86 (0.81–0.89)	0.84 (0.80–0.87)	0.91 (0.85–0.95)*^,^^§§§^
Glucose (mmol/L)	4.6 (4.2–5.0)	4.9 (4.5–5.3)*	4.4 (4.1–5.1)
HbA1c (%)	5.1 (4.9–5.4)	5.2 (5–5.45)	5.4 (5.05–5.6)*
Hb (g/dL)	13.5 (12.8–14.4)	13.1 (11.9–14.1)	12.6 (11.6–13.9)*
Urine albumin/creatine ratio (mg/mmol creatinine)	0.56 (0.375–0.925)	0.9 (0.4–2.2)	1.4 (0.47–3.45)*
Blood pressure			
Mean systolic pressure (mmHg)	116 (111–127.5)	117 (111–129.7)	119 (110–133)
Mean diastolic pressure (mmHg)	81 (75.65–91.5)	84.5 (78–91)	86 (76–90)
Mean heart rate (bpm)	67 (61.5–75.5)	68 (62–76)	74 (64–82)*
Lipid profile			
Total cholesterol (mmol/L)	4.22 (3.62–4.47)	4.28 (3.87–4.79)	3.79 (3.22–4.41)^§^
HDL-cholesterol (mmol/L)	1.33 (1.26–1.60)	1.3 (1.05–1.66)	1.05 (0.93–1.35)**^,^^§^
LDL-cholesterol (mmol/L)	2.09 (1.90–2.61)	2.36 (2.03–2.77)	2.24 (1.70–2.63)
Triglycerides (mmol/L)	0.84 (0.67–1.04)	0.88 (0.71–1.16)	0.86 (0.59–1.38)
Lp(a) (U/L)	0.92 (0.53–1.53)	1.33 (0.39–2.31)	1.10 (0.43—1.88)
apoA1 (µmol/L)	45.5 (41–49.125)	43.8 (37.875–51.25)	38.5 (35.6–43.1)**^,^^§^
apoB (µmol/L)	1.47 (1.24–1.78)	1.55 (1.40–1.80)	1.47 (1.15–1.76)
apoB/ apoA1 ratio	0.61 (0.51–0.74)	0.62 (0.51–0.80)	0.69 (0.50–0.85)
Cardiovascular risk estimation			
FMD (%)	7 (5.35–10.82)	6.64 (3.01–9.49)	4.96 (2.6–9.11)
cIMT (µm)	558 (497.25–647.25)	601 (560–698.5)	596.25 (540.5–657)
FRS (%)	2.15 (1.4–3.45)	2.00 (1.45–6.05)	2.10 (1.40–5.20)
Anti-apoA1 IgG OD	0.39 (0.28–0.62)	0.55 (0.38–0.91)***	0.97 (0.63–1.54)****^,^^§§§^
Anti-apoA1 IgG POS prevalence	12/50	20/50	31/44
Inflammatory cytokines			
IFN-gamma (pg/mL)	1.77 (1.28–2.89)	3.5 (1.59–6.51)*	6.49 (4.28–9.93)****^,^^§§§^
IL-10 (pg/mL)	0.13 (0.08–0.19)	0.2 (0.09–0.42)*	0.43 (0.32–0.61)****^,^^§§§§^
IL-6 (pg/mL)	0.37 (0.24–0.54)	0.72 (0.37–1.27)***	0.80 (0.52–1.72)****
IL-8 (pg/mL)	10.91 (7.93–13.45)	14.34 (10.08–19.75)**	11.84 (8.69–16.49)
TNF-alpha (pg/mL)	1.07 (0.89–1.27)	1.55 (1.09–2.39)***	3.04 (2.43–4.67)****^,^^§§§§^
MCP-1 (pg/mL)	998.9 (767.2–1,281)	1,017 (657.1–1,396)	1,069 (762.5–1,590)
MIP-1 alpha (pg/mL)	30.87 (19.49–43.98)	46.32 (31.21–64.72)**	56.21 (42.99–70.99)****
MIP-1 beta (pg/mL)	415.5 (298.1–582.9)	412.8 (268.3–590.5)	350.7 (240.1–455.3)
hsCRP (mg/L/L)	4.00 (1.00–8.20)	6.85 (2.72–15.93)*	5.60 (1.10–15.40)
ICAM-1 (pg/L)	425.7 (352.1–473.4)	524.3 (429.4–681.5)***	617.4 (487.9–771.7)****
VCAM-1 (pg/L)	453.6 (391.2–541)	536.7 (484.3–688.3)***	742.1 (630.8–966.7)****^,^^§§§^
SAA (pg/L)	1,329 (871.8–6,202)	1,911 (659.2–8,476)	2,713 (990.5–15,175)
Tryptophan pathway metabolites			
L-Tryptophan (AUC)	49,132 (45,299–53,124)	44,162 (35,601–51,621)**	47,616 (43,674–53,269)
Kynurenine (AUC)	4,174 (3,597–5,452)	4,699 (3,869–5,927)	6,416 (5,195–8,195)****^,^^§§^
Kynurenine/L-Tryptophan	0.087 (0.069–0.11)	0.11 (0.08–0.14)*	0.12 (0.10–0.17)****
3-Hydroxykynurenine (AUC)	19.11 (13.88–30.16)	30.14 (18.99–46.88)*	30.19 (17.94–54.11)*
5-Hydroxyindoleacetic acid (AUC)	2,747 (2,214–3,138)	2,824 (2,444–3,624)	3,756 (2,974–4,891)****^,^^§§^
Kynurenic acid (AUC)	2,694 (2,010–5,317)	2,669 (1,649–3,837)	4,934 (2,972–7,883)*^,^^§§^
Indole-3-acetaldehyde (AUC)	74.97 (57.76–92.30)	61.82 (48.07–84.19)	64.47 (51.17–85.54)
Xanthurenic acid (AUC)	668.1 (541.5–820.6)	562.9 (461.9–677.0)*	550.0 (450.2–678.4)**
Indole-3-acetate (AUC)	2,439 (1,919–3,352)	2,012 (1,372–3,163)	2,349 (1,702–4,130)
Quinoldic acid: 2-quinoline carboxylic acid (AUC)	94.35 (58.65–127.7)	89.54 (59.25–128.5)	72.76 (55.18–101.1)

The table is divided into the following groups: control volunteers without HIV (control volunteers), PLWH ART-treated, and PLWH-ART naïve. A list of clinical parameters and inflammatory cytokines is also provided. Values are expressed as medians (interquartile ranges). Statistical difference was evaluated using non-parametric Kruskwal–Wallis tests.

* *p* < 0.05 was considered significant vs. control.

** *p* < 0.01 was considered significant vs. control.

*** *p* < 0.001 was considered significant vs. control.

**** *p* < 0.0001 was considered significant vs. control.

^§^ *p* < 0.05 was considered significant between PLWH ART-treated and PLWH ART-naïve.

^§§^ *p* < 0.01 was considered significant between PLWH ART-treated and PLWH ART-naïve.

^§§§^ *p* < 0.001 was considered significant between PLWH ART-treated and PLWH ART-naïve.

^§§§§^ *p* < 0.0001 was considered significant between PLWH ART-treated and PLWH ART-naïve.

Despite the trends observed in the biochemical variables and biomarkers, which were overall suggestive of a pro-atherogenic profile, there were no differences in FRS, cIMT, and FMD between the three groups.

### Correlation between FRS and cIMT

**Figure 2:**
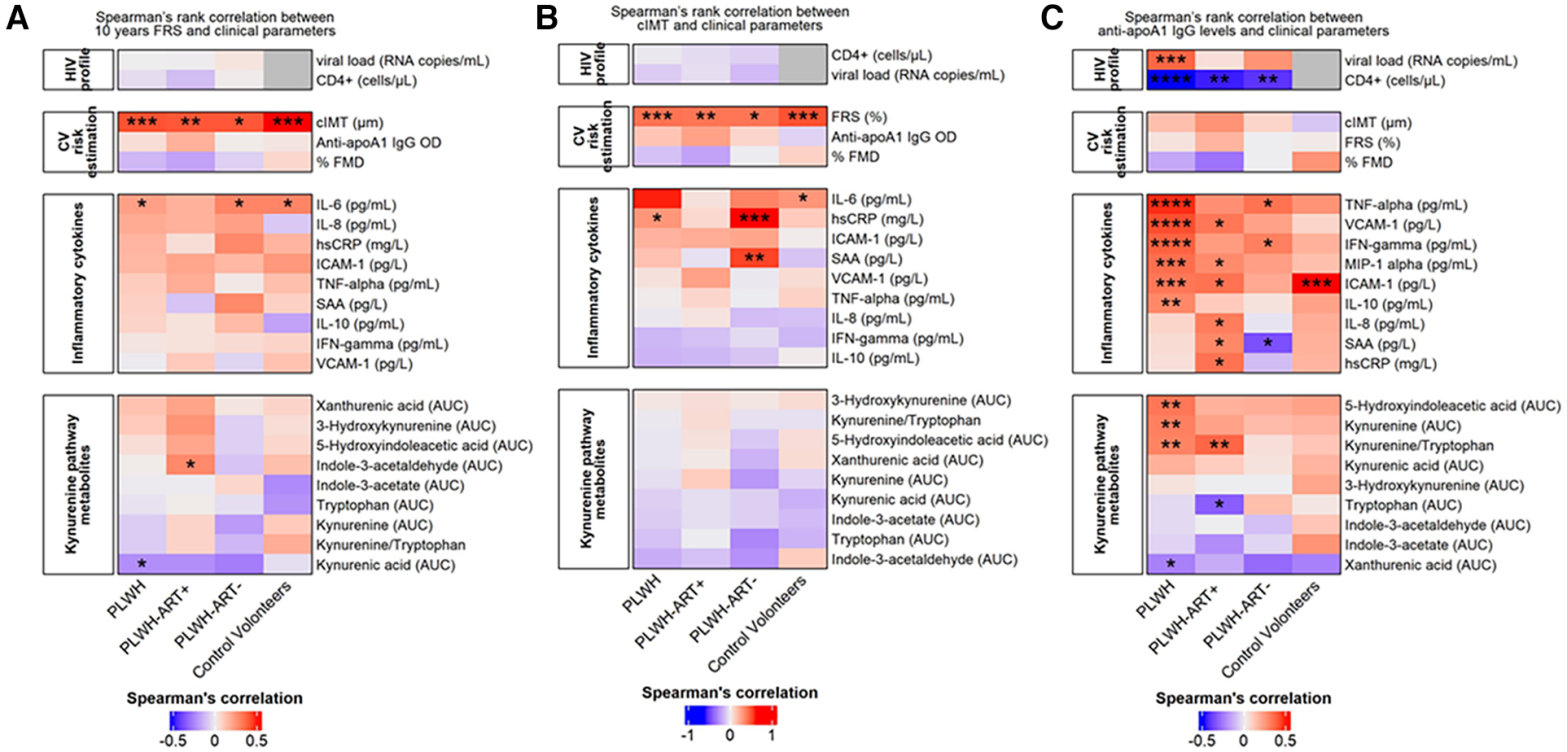
Cardiovascular risk estimation by Spearman's rank correlation. (**A**) Spearman's rank correlation between the FRS and clinical parameters. The heatmap is divided into subject groups of PLWH (*n* = 94), PLWH ART-treated (PLWH-ART+, *n* = 50), PLWH ART-naïve (PLWH-ART−, *n* = 44), and control volunteers (*n* = 49). (**B**) Spearman's rank correlation between cIMT and clinical parameters. The heatmap is divided into subject groups of PLWH (*n* = 74), PLWH ART-treated (PLWH-ART+, *n* = 46), PLWH ART-naïve (PLWH-ART−, *n* = 28), and control volunteers (*n* = 48). (**C**) Spearman's rank correlation between anti-apoA1 IgG and clinical parameters. The heatmap is divided into subject groups of PLWH (*n* = 94), PLWH ART-treated (PLWH-ART+, *n* = 50), PLWH ART-naïve (PLWH-ART−, *n* = 44), and control volunteers (*n* = 49). FRS, cIMT, and anti-apoA1 IgG were correlated to the following categories: HIV profile, CV risk estimation, inflammatory cytokines, and kynurenine pathway metabolites. In each category, parameters were top-down ranked according to the *r* value. Statistic difference was evaluated using non-parametric Spearman ranking tests: * *p*-values <0.05, ** < 0.01, *** < 0.001, and **** < 0.0001 were considered significant.

Spearman correlations indicated positive and significant correlations between cIMT and FRS across all three subgroups (*r* ranging between 0.41 and 0.51), but no correlation was observed between FMD and FRS (Figure 2).

Except for IL-6, which modestly correlated with the FRS across the three groups, no significant associations between the other cytokines and the FRS were observed (Figure 2A). Similarly, no associations were found between FRS and tryptophan pathway metabolites (Figure 2A) or between cIMT and cytokines or tryptophan pathway metabolites (Figure 2B).

### Anti-apoA1 IgG levels across study groups and associations with inflammatory cytokines and tryptophan pathway metabolites

As shown in Table 1, participants with HIV displayed higher median anti-apoA1 IgG levels and seropositivity rates than healthy volunteers, while PLWH ART-naïve displayed the highest values and seropositivity rates. The association of anti-apoA1 IgG with the different parameters was analysed in the three groups. Anti-apoA1 IgG was not associated with FRS, cIMT, or FMD, despite a possible trend with FRS and cIMT in the PLWH ART-treated group (*p* = 0.061 and *p* = 0.083, respectively) (Table 2).

**Table 2:** Difference between anti-apoA1 IgG POS and anti-apoA1 IgG NEG in the different group populations.

Parameters	HIV-free anti-apoA1 NEG (*n* = 37)	HIV-free anti-apoA1 POS (*n* = 12)	PLWH anti-apoA1 NEG (*n* = 43)	PLWH anti-apoA1POS (*n* = 51)	PLWH ART-treated anti-apoA1NEG (*n* = 30)	PLWH ART-treated anti-apoA1POS (*n* = 20)	PLWH ART-naïve anti-apoA1NEG (*n* = 13)	PLWH ART-naïve anti-apoA1POS (*n* = 31)
Metabolic parameters								
BMI	23.10	20.89	22.40	20.10	22.65	19.80*	20.59	20.70
HIV profile								
CD4+ (cells/µL)			552	414**	554	391**	526	444
Viral load (RNA copies/mL)			51	7,836*	27.5	15	25,047	24,249
Lipid profile								
Triglycerides (mmol/L)	0.84	0.90	0.88	0.86	0.88	0.96	1.10	0.77
Total cholesterol (mmol/L)	4.22	4.16	4.23	3.88	4.46	4.16	3.78	3.79
HDL-C (mmol/L)	1.32	1.33	1.15	1.14	1.47	1.25	0.99	1.09
LDL-C (mmol/L)	2.03	2.32	2.38	2.24	2.48	2.23	2.09	2.25
apoB/apoA1	0.607	0.618	0.622	0.677	0.615	0.626	0.673	0,692
Cardiovascular risk estimation								
FRS (%)	0.35	0.30	0.2	0.5	0.2	1.3	0.7	0.3
FMD (%)	3.457	3.551	3.336	3.186	3.395	3.366	3.011	3.163
cIMT (μm)	589	520.3	593.5	648	599	680.5	586.5	613.3
Inflammatory cytokines								
INFγ (pg/mL)	1.74	1.795	2.910	5.16**	2.72	4.35	5.53	7.21
IL-6 (pg/mL)	0.33	0.36	0.67	0.87	0.62	0.975**	1.01	0.66
IL-8 (pg/mL)	10.53	12.82	11.71	13.46	11.85	18.71*	11.71	11.89
TNF (pg/mL)	1.01	1.24	1.69	2.9***	1.39	1.99*	2.68	3.26
MIP1 alpha (pg/mL)	30.17	31.19	46.79	56.66**	40.67	49.73	49.14	63.55
hsCRP (mg/L)	2,823	7,772*	4,382	5,952	4,011	13,324**	14,947	4,137
ICAM-1 (pg/L)	416.6	506.1**	515.6	637.8*	490	640.7*	582.6	637.8
VCAM-1 (pg/L)	463.6	446.5	537.4	714.7**	520.1	571.7*	728.2	767.5
SAA (pg/L)	1,153	9,396**	1,829	2,608	1,557	7,085*	8,104	2,233*
Tryptophan pathway metabolites								
L-Tryptophan (AUC)	48,775.5	49,910.3	45,191.9	47,271.4	45,111.7	41,359.4*	45,191.9	47,666.7
Kynurenine (AUC)	4,028.5	5,374.4**	4,883.4	5,875.5*	4,355.0	5,145.8	7,041.0	6,312.3
Kynurenic acid (AUC)	2,628.1	2,693.7	2,678.8	4,058.0*	2,232.6	3,604.4	4,911.3	5,175.8
Indole-3-alcetaldehyde (AUC)	74.5	77.2	63.2	59.6	473.6	591.0	76.4	57.5
Xhanturenic acid (AUC)	712.0	595.9	596.0	525.7	562.9	536.2	631.5	525.7*
Indole-3-acetate (AUC)	2,282.4	3,135.4*	2,444.8	1,974.9	2,367.6	1,790.4	2,519.2	2,330.1
5-hydxoxyindolacetate (AUC)	2,638.8	3,600.2**	2,855.8	3,497.5*	2,762.3	3,002.3	3,312.7	3,911.3
3-hydroxykyunerinie (AUC)	18.83	27.02	28.79	32.57	28.94	36.72	22.31	30.86
Kynurenine/L-Tryptophan	0.079	0.108	0.104	0.124*	0.101	0.122**	0.155	0.126

The table is divided into the following groups: HIV-free, PLWH, PLWH ART-treated, and PLWH ART-naïve. A list of clinical parameters, inflammatory cytokines, and tryptophan pathway metabolites is provided. Values are expressed as medians. Statistical difference was evaluated using non-parametric Mann–Whitney tests.

* *p* < 0.05 was considered significant between anti-apoA1 IgG-positive and -negative patients.

** *p* < 0.01 was considered significant between anti-apoA1 IgG-positive and -negative patients.

*** *p* < 0.001 was considered significant between anti-apoA1 IgG-positive and -negative patients.

**Figure 3:**
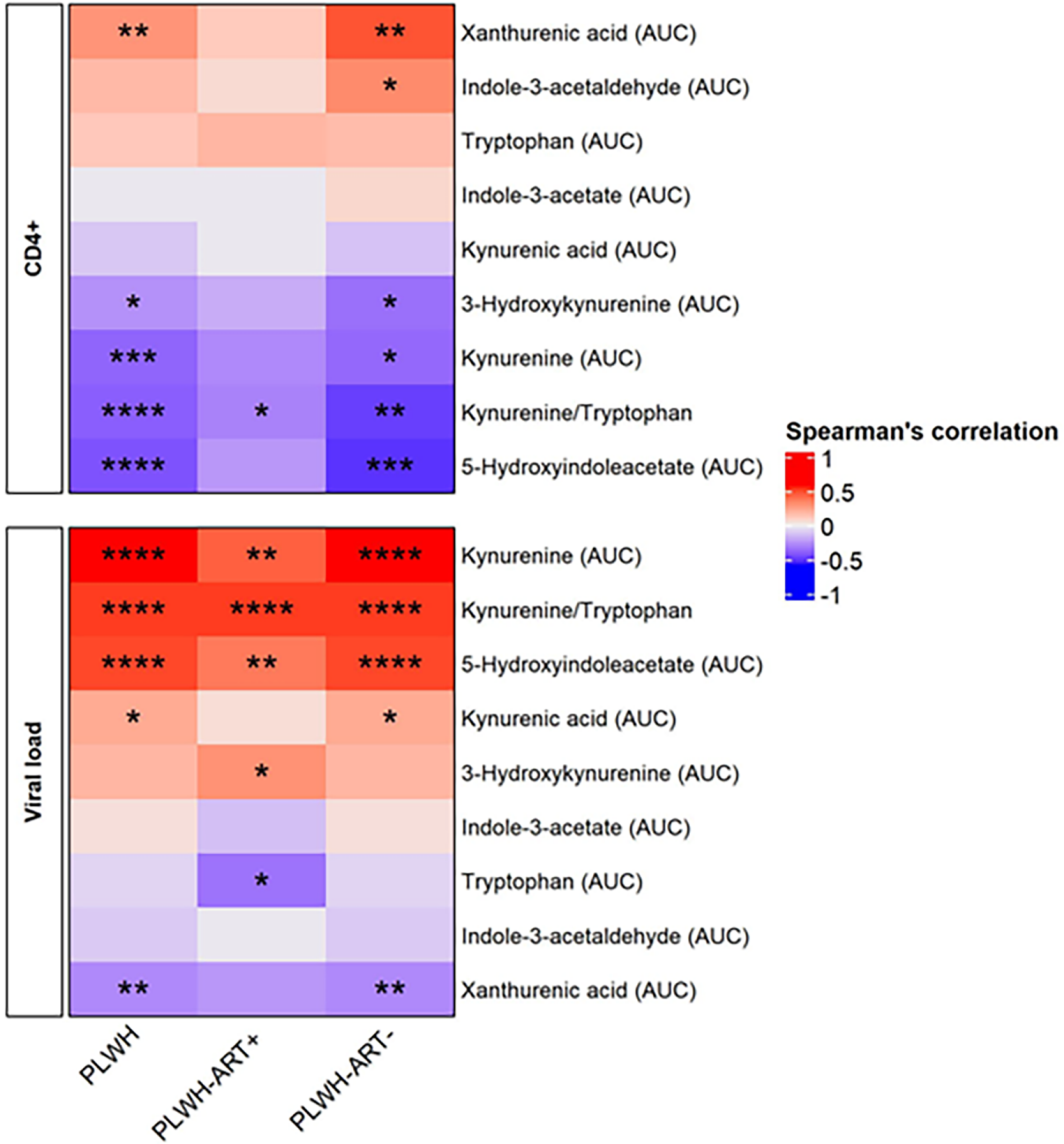
Spearman's rank correlation between the HIV profile and kynurenine pathway metabolites. The heatmap is divided into subjects groups of PLWH (*n* = 94), PLWH ART-treated (PLWH-ART+, *n* = 50), PLWH ART-naïve (PLWH-ART−, *n* = 44) and by the following parameters: CD4+ cell count (cells/µL) and viral load (RNA copies/mL). In each category, parameters were top-down ranked according to the *r* value. Statistic difference was evaluated using non-parametric Spearman ranking tests: * *p*-values <0.05, ** < 0.01, *** < 0.001, and **** < 0.0001 were considered significant.

When dichotomising the three study groups according to anti-apoA1 IgG seropositivity status, we observed that HIV-negative anti-apoA1 IgG seropositive participants had higher circulating median hsCRP, ICAM-1, SAA, kynurenine, indole-3-acetate, and 5-hydxoxyindolacetate levels compared to seronegative participants (Table 2). Among PLWH, anti-apoA1 IgG seropositive individuals displayed higher median values of INFγ, TNFα, MIP1alpha, ICAM-1, VCAM-1, kynurenine, kynurenic acid, 5-hydxoxyindolacetate, and kynurenine/tryptophan ratio than seronegative ones. Anti-apoA1 IgG seropositive individuals displayed lower CD4+ cell counts and higher viral load than seronegative individuals. Despite limited statistical power, we further explored the unique study group consisting of PLWH ART-naïve to better understand the possible contribution of ART to inflammation, the kynurenine pathway, and cardiovascular phenotype. In the PLWH ART-treated group, anti-apoA1 IgG was positively correlated with IL-8, hsCRP, ICAM-1, VCAM-1, and SAA, while in the PLWH ART-naïve group, anti-apoA1 IgG was positively correlated with INFγ and TNFα and inversely correlated with SAA (Figure 2C). Regarding the tryptophan/kynurenine pathway metabolites, the kynurenine/tryptophan ratio and xanthurenic acid were increased and decreased, respectively, in PLWH anti-apoA1 IgG seropositive participants (Table 2). The tryptophan pathway metabolites were associated with HIV parameters. While CD4+ cell counts were positively correlated with xanthurenic acid and negatively correlated with kynurenine, 5-hydroxyindoleacetate, 3-hydroxykynurenine, and kynurenine/tryptophan ratio, the viral load was positively correlated with kynurenine, 5-hydroxyindoleacetate, and kynurenic acid and negatively correlated with xanthurenic acid (Figure 3). Of note, ART affected the levels of kynurenine, kynurenine/tryptophan ratio, 5-hydroxindoleacetate, kynurenic acid, and xanthurenic acid. Interestingly, xanthurenic acid was associated with anti-apoA1 IgG seropositivity in PLWH ART-naïve and HIV parameters (Table 2 and Figure 3). In addition, in the HIV ART-treated group, anti-apoA1 IgG seropositive patients displayed a significantly lower body mass index (Table 2).

### Anti-apoA1 IgG and adhesion molecules in HAECs

**Figure 4:**
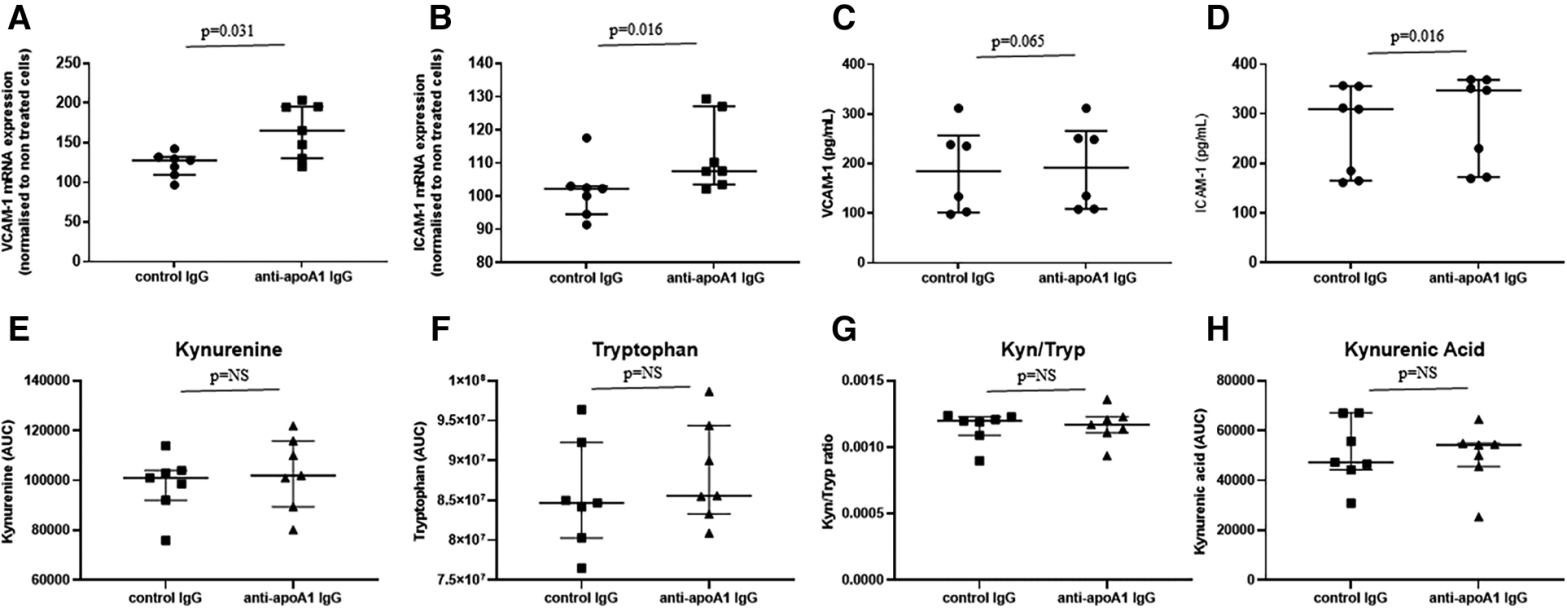
Anti-apoA1 IgG treatment induces ICAM-1 and VCAM-1 expression but not tryptophan pathway metabolites. HAECs were incubated with anti-apoA1 IgG (40 µg/mL) or control IgG (40 µg/mL) for 24 h. Cells were lysed, mRNA was extracted, and the expression of VCAM-1 and ICAM-1 was analysed (**A,B**). The incubation medium was collected and the levels of VCAM-1 and ICAM-1 were quantified using the MSD platform (**C,D**). The incubation medium was collected, and the levels of kynurenine, tryptophan, kynurenine/tryptophan ratio (Kyn/Tryp), and kynurenic acid were quantified using LC/MRM-MS (**E–H**). Results are expressed as medians with interquartile ranges. Statistical difference was evaluated using non-parametric Wilcoxon tests; *p* < 0.05 was considered significant.

To identify a possible causal link between higher levels of circulating adhesion molecules (ICAM-1 and VCAM-1) and some metabolites of the tryptophan pathway, we evaluated *in vitro* the impact of anti-apoA1 IgG stimulation on VCAM-1 and ICAM-1 production on HAECs, as well as the levels of tryptophan metabolites in the cell supernatant. HAECs were incubated for 24 h with polyclonal anti-apoA1 IgG or the isotype control antibody (40 µg/mL), followed by the measurement of the ICAM-1 and VCAM-1 intra-cellular mRNA expression levels as well as their presence in the cell supernatant. The results indicated that anti-apoA1 IgG treatment of HAECs induced the mRNA expression as well as the release into the medium of ICAM-1 and VCAM-1 *in vitro* (Figures 4A–D). In the same supernatant, the levels of kynurenine, tryptophan, and kynurenic acid were measured, and we did not observe a significant change in tryptophan, kynurenine, kynurenine/tryptophan ratio, or kynurenic acid upon anti-apoA1 IgG stimulation (Figures 4E–H).

## Discussion

The first important finding of this study is the confirmation that HIV infection increases the humoral autoimmune response against apoA1 in our study population. Consistent with previous observations indicating that RNA virus infections, such as hepatitis C, SARS-CoV2, and HIV, induce an anti-apoA1 IgG response (18–20), this study confirms preliminary results suggesting that such biological signature is associated with canonical biological determinants of HIV severity, including higher viremia and lower CD4+ cell counts, which are known to be major determinants of cardiovascular complications in PLWH (37, 38). The unique design of this study allowed us to demonstrate an anti-apoA-1 IgG seropositivity (expressed by anti-apoA-1 IgG positivity prevalence) gradient across the three study groups, starting at 24% in healthy volunteers, 54% in PLWH ART-treated, and reaching 70% in PLWH ART-naïve. Although no causal relationship can be inferred from our data, a previous study showed that anti-apoA1 IgG could promote CD4+ cell apoptosis and elicit a proatherogenic response by promoting inflammation, foam cell formation, and myocardial necrosis in a TLR2/TLR4/CD14-dependent manner (19, 21, 23, 26). Despite the inconsistent data regarding ART and cardiovascular risk, the cardiovascular benefits of early ART are nowadays recognised to offset their potential intrinsic cardiovascular hazards by effectively controlling the exaggerated pro-inflammatory and pro-coagulant states in individuals living with HIV (31). The lower rate of anti-apoA1 seropositivity observed in ART-treated individuals is in line with the proposed pro-atherogenic properties of anti-apoA1 IgG. In addition, anti-apoA1 IgG could directly promote the endothelial expression of ICAM-1 and VCAM-1. Although the present study did not investigate underlying molecular mechanisms, previous publications showed that ICAM-1 and VCAM-1 expression is TLR2/TLR4-dependent (39–41), suggesting that the same innate immune receptors could be involved in anti-apoA1 IgG-induced trans-endothelial migration of immunocompetent cells. The impact of ART on auto-antibodies is poorly documented; however, the observation in our study is in line with the findings of Marinho and colleagues (42), who showed that the anti-retroviral nevirapine (NVP) was able to lower the titres of anti-HDL auto-antibodies, which are closely related to anti-apoA1 antibodies. However, since only one participant in the current study cohort was treated with NVP, we could not replicate this observation with anti-apoA1 IgG.

Importantly, we could not detect any significant association between these auto-antibodies and the FRS, cIMT, or FMD. This is in contrast with previous data derived from other populations (43, 44). The inconsistency could be explained by several mutually non-exclusive reasons, including differences in study populations and statistical power, variability in imaging diagnostic modalities, the fact that these antibodies seem to behave as independent cardiovascular risk factors in the general population (15), or because these antibodies have mostly been associated with atherosclerotic plaque vulnerability rather than the size of atherosclerotic lesions (23).

The second important finding of the present study is the identification of significant correlations between anti-apoA1 IgG and tryptophan metabolism, showing that the auto-antibodies are associated with increased activation of the tryptophan pathway, characterised by lower tryptophan levels and higher kynurenine metabolite levels, resulting in a higher kynurenine/tryptophan ratio. Although our *in vitro* experimental approach using HAECs may not be regarded as an optimal model to demonstrate a possible causal association between anti-apoA1 IgG and the kynurenine pathway, it is, to our knowledge, the first report linking anti-apoA1 IgG antibodies to this pathway. Further experimental work on various immunocompetent cells is required to determine whether anti-apoA1 IgG could directly modulate the activation of indoleamine 2,3-dioxygenase (IDO) enzyme required to convert tryptophan to kynurenine (45). This will help to better delineate the respective contributions of anti-apoA1 IgG and kynurenine in VCAM-1/ICAM-1 expression, as IDO inhibition has been shown to increase atherosclerotic lesions size and upregulation of VCAM-1 (45).

Activation of the tryptophan catabolism pathway has been implicated in the pathogenesis of various processes, including, among others, coronary artery disease (46) and HIV; increased levels of various tryptophan metabolite levels were found to be associated with HIV serostatus, severity of HIV infection, low-grade inflammation in HIV-positive individuals with virological suppression, and long-term non-AIDS-related events (47), as well as being modulated by ART (48–51). The significant correlations observed in the present study between kynurenine and the kynurenine/tryptophan ratio, CD4+ cell counts, and viral load results are in line with these observations, as well as the fact that ART was associated with lower levels of kynurenine and the kynurenine/tryptophan ratio. In this study, we extended the list of kynurenine metabolites associated with HIV parameters and/or ART with additional kynurenine metabolites, viz., xanthurenic acid, 5-hydroxyindoleacetate, 3-hydroxykynurenine, and kynurenic acid.

The fact that the levels of tryptophan pathway metabolites were not associated with an increased FRS nor with higher atherosclerosis burden measures (cIMT, FMD) contrasts with other studies demonstrating a correlation between tryptophan metabolites and cIMT (52, 53). This divergence can certainly be partly explained by an age difference between our study and others (median age of 35 years in our study vs. >42 years), although other factors cannot be excluded (52, 53).

The first limitation of our study relates to the fact that, despite significant correlations retrieved between cIMT and FRS across all three subgroups, no inter-group differences in the FRS (cIMT and FMD) were observed. Although this is most likely explained by relatively small sample sizes and the young age of the included participants (median: 35 years old), the absence of inter-group difference in terms of cardiovascular risk or atherosclerosis-related burden renders the interpretation of the current correlations with CVD rather difficult. Another limitation resides in the fact that the kynurenine pathway functionality in endothelial cells is still debated, and experiments on immunocompetent cells are required before formally concluding the absence of a causal association between anti-apoA1 IgG and kynurenine pathway activation. Moreover, we did not measure other auto-antibodies, such as anti-HDL antibodies. Given that anti-apoA1 IgG is the most extensively studied and validated, we focused on this specific class of auto-antibodies rather than those ascribed to a “broader” HDL autoimmune response, including antibodies against HDL, against LCAT and against-PON-1 (25). Finally, while demonstrating that anti-apoA1 IgG induced ICAM-1 and VCAM-1, we hypothesised that TLR2/TLR4 pathway activation explains our current results, corroborating and extending the pro-atherogenic properties of anti-apoA1 IgG reported previously, probably through the same mechanism (19, 22, 26). In fact, VCAM-1/ICAM-1 expression is mediated by TLR2/TLR4, and subsequent identical intra-cellular pathway activation is reported (40, 54). We neither investigate the association between persistent HIV proteins (such as Nef, Tat, and gp120 proteins), known to potentially induce endothelial dysfunction by promoting apoptosis, inflammatory cytokines, and ICAM-1/VCAM-1 expression (55–57), and the levels of anti-apoA1 IgG and kynurenine pathway metabolites nor the other parameters measured in this study. Further investigations would be necessary to elucidate the precise mechanism of action of anti-apoA1 IgG on endothelial cells; however, such investigations fall outside the scope of the present study.

In conclusion, our data highlighted that HIV infection increases the humoral response against apoA1 in our study cohort, an effect that is associated with biological features of HIV severity. We also demonstrated that these auto-antibodies may induce ICAM-1 and VCAM-1 expression in endothelial cells. Furthermore, this work delineates, for the first time, an intimate relationship between anti-apoA1 IgG and kynurenine pathway activation, whose pathophysiological relevance is broad and covers HIV, autoimmunity, and atherosclerosis pathogenesis. Whether anti-apoA1 IgG could directly modulate kynurenine pathway activation and be used as a biomarker to predict incident cardiovascular events in PLWH remains to be demonstrated.

## Data Availability

The original contributions presented in the study are included in the article/Supplementary Material; further inquiries can be directed to the corresponding author.
